# Investment case for improving maternal and child health: results from four countries

**DOI:** 10.1186/1471-2458-13-601

**Published:** 2013-06-21

**Authors:** Eliana Jimenez Soto, Sophie La Vincente, Andrew Clark, Sonja Firth, Alison Morgan, Zoe Dettrick, Prarthna Dayal, Bernardino M Aldaba, Soewarta Kosen, Aleli D Kraft, Rajashree Panicker, Yogendra Prasai, Laksono Trisnantoro, Beena Varghese, Yulia Widiati

**Affiliations:** 1 School of Population Health, 4th Floor, Public Health Building, University of Queensland, Herston Road, Herston, QLD 4006, Australia; 2 Centre for International Child Health, Murdoch Childrens Research Institute, Level 4, Front Entry Building, Royal Children's Hospital, University of Melbourne, Flemington Road, Parkville, VIC 3052, Australia; 3 Department of Health Services Research and Policy, London School of Hygiene & Tropical Medicine, 15-17 Tavistock Place, London WC1H 9SH, UK; 4 Nossal Institute for Global Health, Level 4, Alan Gilbert Building, University of Melbourne, 161 Barry Street, Carlton, VIC, 3010, Australia; 5 UPecon Foundation, Inc. Room 322, UP School of Economics, University of the Philippines, Diliman, Quezon City 1101, Philippines; 6 Center for Health Systems and Policy Research & Development, National Institute of Health Research & Development, Jalan Percetakan Negara 23A, Jakarta 10560, Indonesia; 7 Public Health Foundation of India (PHFI) 4, Institutional Area, Vasant Kunj, New Delhi 110 070, India; 8 New ERA, Rudramati Marg, Kalo Pul, Kathmandu, Nepal; 9 Centre for Health Service Management, Faculty of Medicine, Gadjah Mada University, Jl. Farmako Sekip Utara, Jogjakarta 55281, Indonesia

**Keywords:** Maternal, Newborn and Child Health, Evidence-based Planning, Scaling-up Interventions, Equity

## Abstract

**Background:**

Without addressing the constraints specific to disadvantaged populations, national health policies such as universal health coverage risk increasing equity gaps. Health system constraints often have the greatest impact on disadvantaged populations, resulting in poor access to quality health services among vulnerable groups.

**Methods:**

The Investment Cases in Indonesia, Nepal, Philippines, and the state of Orissa in India were implemented to support evidence-based sub-national planning and budgeting for equitable scale-up of quality MNCH services. The Investment Case framework combines the basic setup of strategic problem solving with a decision-support model. The analysis and identification of strategies to scale-up priority MNCH interventions is conducted by in-country planners and policymakers with facilitation from local and international research partners.

**Results:**

Significant variation in scaling-up constraints, strategies, and associated costs were identified between countries and across urban and rural typologies. Community-based strategies have been considered for rural populations served predominantly by public providers, but this analysis suggests that the scaling-up of maternal, newborn, and child health services requires health system interventions focused on 'getting the basics right'. These include upgrading or building facilities, training and redistribution of staff, better supervision, and strengthening the procurement of essential commodities. Some of these strategies involve substantial early capital expenditure in remote and sparsely populated districts. These supply-side strategies are not only the 'best buys', but also the 'required buys' to ensure the quality of health services as coverage increases. By contrast, such public supply strategies may not be the 'best buys' in densely populated urbanised settings, served by a mix of public and private providers. Instead, robust regulatory and supervisory mechanisms are required to improve the accessibility and quality of services delivered by the private sector. They can lead to important maternal mortality reductions at relatively low costs.

**Conclusions:**

National strategies that do not take into consideration the special circumstances of disadvantaged areas risk disempowering local managers and may lead to a “business-as-usual” acceptance of unreachable goals. To effectively guide health service delivery at a local level, national plans should adopt typologies that reflect the different problems and strategies to scale up key MNCH interventions.

## Background

The difficulties inherent in scaling-up proven interventions to reduce maternal, newborn and child mortality will mean that many countries will fail to hit their targets for MDGs 4 & 5 [1,2]. The issues to consider for prioritisation and scale-up of interventions are well documented [3,4] and costs and impact of a broad scale up of key interventions to a prescribed coverage level across countries have been estimated [5]. Such reviews and studies are useful for providing broad recommendations and information to countries on scaling up interventions at a national level. However they fail to capture the specific problems associated with scaling up services to disadvantaged communities, which need to be addressed to avoid countries increasing the equity gap [4].

The Investment Case (IC) was launched by development partners in the Asia-Pacific region with the goal to support in-country implementation and more equitable outcomes for maternal, newborn and child health (MNCH). As part of this initiative, we have worked with planners and policymakers in four diverse Asian countries (Indonesia, Nepal, the Philippines, and the State of Orissa in India) to formulate and cost locally relevant strategies to scale up MNCH services in disadvantaged settings. The Investment Case framework combines the basic setup of strategic problem solving [6] with a decision-support model. The key steps in the problem-solving analysis and the implications and lessons learnt of this approach to inform locally produced plans and budgets are presented elsewhere [7]. In this paper we focus on the results of this analysis as they pertain to a select number of disadvantaged districts and cities in our study countries.

## Methods

### The setting

#### Selection of sites

The importance of geography as both an equity marker [8] and the key parameter used by governments for planning, budgeting and delivery of health services justified the choice of disadvantaged locations as the unit of analysis. Time and logistical constraints prevented us from undertaking this work in a large number of sites. With the understanding that many health system constraints are by nature common across sites with similar typologies, policymakers in devolved settings opted to pilot Investment Cases in a few locations representative of “typical” disadvantaged sub-national units. Different criteria were used in each country. In the Philippines, two provinces from the Eastern Visayas region with high mortality were chosen. Due to the concerns of policymakers with the urban-poor, a city was also included in the analysis. In Indonesia, two rural and two urban sites with poor coverage of MNCH interventions were selected. In Orissa, government officials chose two districts with large disadvantaged populations, one predominantly tribal and one prone to natural disasters. In the centralised health system of Nepal, the reality of health services delivery dictates that the strategies needed to scale up services in the populous plains (terai) regions, are different to those required for the hills and remote mountainous districts. Therefore groups of districts with low intervention coverage were chosen, which were representative of the different ecological regions. Table 1 provides key characteristics and demographics of the sites selected in each country.

**Table 1:** Key characteristics and demographics of investment case study sites

**Characteristics**	**Philippines**			**Indonesia**				**India (Orissa state)**		**Nepal**		
	**Northern Samar Province**	**Eastern Samar Province**	**Pasay City**	**Sikka District**	**Meruake District**	**Tasikmalaya City**	**Pontianak City**	**Kendrapara District**	**Rayagada District**	**Terai Cluster**	**Hills Cluster**	**Mountain Cluster**
Description	Rural province in Philippines, with poor MNCH Outcomes and low fiscal capacity. Limited availability of delivery facilities and existing facilities are poorly supplied. Large proportion of births occurs unassisted at home.	Rural province in Philippines, with poor MNCH Outcomes, higher fiscal capacity and lower population than Northern Samar. Limited availability of delivery facilities and existing facilities are poorly supplied. Large proportion of births occurs unassisted at home.	Urban city in Philippines with relatively low mortality, but high levels of inequity in access. Large number of private facilities, but concerns about quality of care. Heavy load on public facilities from most disadvantaged population.	Rural district on coast of East Nusa Tenggara province. Government has low fiscal capacity; population itself has low levels of education and high levels of poverty. ~10% of population live on isolated islands. Malaria is endemic.	Rural district within Papua Province. Very remote with a high cost of living and limited access to clean water. ~50% of population live in difficult to access mountainous regions. Malaria is endemic.	Urban city within West Java province with a very high population density. Government has low fiscal capacity, and a significant private sector exists. Traditional birth attendants still account for notable proportion of births.	Capital City of West Kalimantan province. Large private sector, with significant number of private midwives. Health knowledge of population is poor, and levels of vaccination have dropped due to recent scare involving adverse effects.	Rural, but not remote, coastal district in Orissa. Poor, with ~67% considered to have a low standard of living. Climatically vulnerable, with access to health services impeded on a seasonal basis. Considered typical of rural districts in coastal areas of Orissa.	Remote, heavily forested tribal district in Orissa. Poor, with ~88% of population considered to have low standard of living. Sparse population and security issues inhibit access to health services. Malaria is endemic. Considered typical of tribal areas of Orissa.	Cluster of disadvantaged districts within the Terai ecoregion. More densely populated than other ecoregions, with fewer access problems.	Cluster of disadvantaged districts within the Hills ecoregion. Significant impact of ten year civil conflict in this cluster	Cluster of disadvantaged districts within Mountain ecoregion. Sparsely populated, with many areas only accessible by air or foot.
Population	670000	440000	410000	300000	192000	642000	522000	1410000	820000	5680000	2340000	860000
	(1)	(1)	(2)	(3)	(4)	(5)	(6)	(7)	(7)	(8)	(8)	(8)
MMR (per 100 000 live births)	160	160	80	228	228	228	228	303	303	281	281	281
	(9)Provincial estimate	(10)Provincial estimate	(2)City estimate	(11)National estimate	(11)National estimate	(11)National estimate	(11)National estimate	(12)State estimate	(12)State estimate	(13)National estimate	(13)National estimate	(13)National estimate
NMR (per 1000 live births)	22	22	17	31	24	19	23	45.4	45.4	26	54	74
	(14)Region 8 estimate	(14)Region 8 estimate	(2)City estimate	(11)Provincial estimate	(11)Provincial estimate	(11)Provincial estimate	(11)Provincial estimate	(15)State estimate	(15)State estimate	(13)Cluster estimate	(13)Cluster estimate	(13)Cluster estimate
U5MR (per 1000 live births)	68	43	28	80	64	59	49	90.6	90.6	89.3	110	168.5
	(9)Provincial Estimate	(16)Provincial estimate	(2)City estimate	(11)Provincial estimate	(11)Provincial estimate	(11)Provincial estimate	(11)Provincial estimate	(15)State estimate	(15)State estimate	(13)Cluster estimate	(13)Cluster estimate	(13)Cluster estimate

Sources:

1. National Epidemiology Center. Field Health Service Information System Annual Report. Manila, Philippines: Department of Health2007.

2. Pasay City Health Office. Pasay City Vital Statistics. Manila, Philippines: Department of Health2008.

3. BPS - Kabupaten Sikka. 2008 Population Registration: BPS-Statistics Indonesia,2008.

4. BPS - Kabupaten Meruake. 2008 Population Registration: BPS-Statistics Indonesia,2008.

5. BPS - Kota Tasik. 2008 Population Registration: BPS-Statistics Indonesia,2008.

6. BPS - Kota Pontianak. 2008 Population Registration: BPS-Statistics Indonesia,2008.

7. Census of India. 2001; Available from: http://www.censusindia.gov.in.

8. HMG Nepal, National Planning Commission Secretariat, Central Bureau of Statistics (CBS); UNFPA. Population Census 2001, National Report. 2002.

9. North Samar Provincial Health Office. Annual Report. Manila, Philippines: Department of Health2008.

10. East Samar Provincial Health Office. Maternal Death Review. Manila, Philippines: Department of Health2009.

11. Statistics Indonesia (Badan Pusat Statistik-BPS) and Macro International. Indonesia Demographic and Health Survey 2007. Calverton, Maryland, USA:: BPS and Macro International.2008.

12. Sample Registration System, Office of the Registrar General of India. Special Bulletin on Maternal Mortality in India 2004–062009.

13. Ministry of Health and Population, New ERA, ORC Macro International Inc. Nepal Demographic and Health Survey 20062007.

14. National Statistics Office. Philippines National Demographic and Health Survey 2008. Manila, Philippines2009.

15. International Institute for Population Sciences (IIPS). National Family Health Survey (NFHS-3) 2005–06, India. 2007;1(1–540).

16. East Samar Provincial Health Office. Annual Report. Manila, Philippines: Department of Health2008.

### The design of the study

#### Problem solving workshops

**Figure 1:**
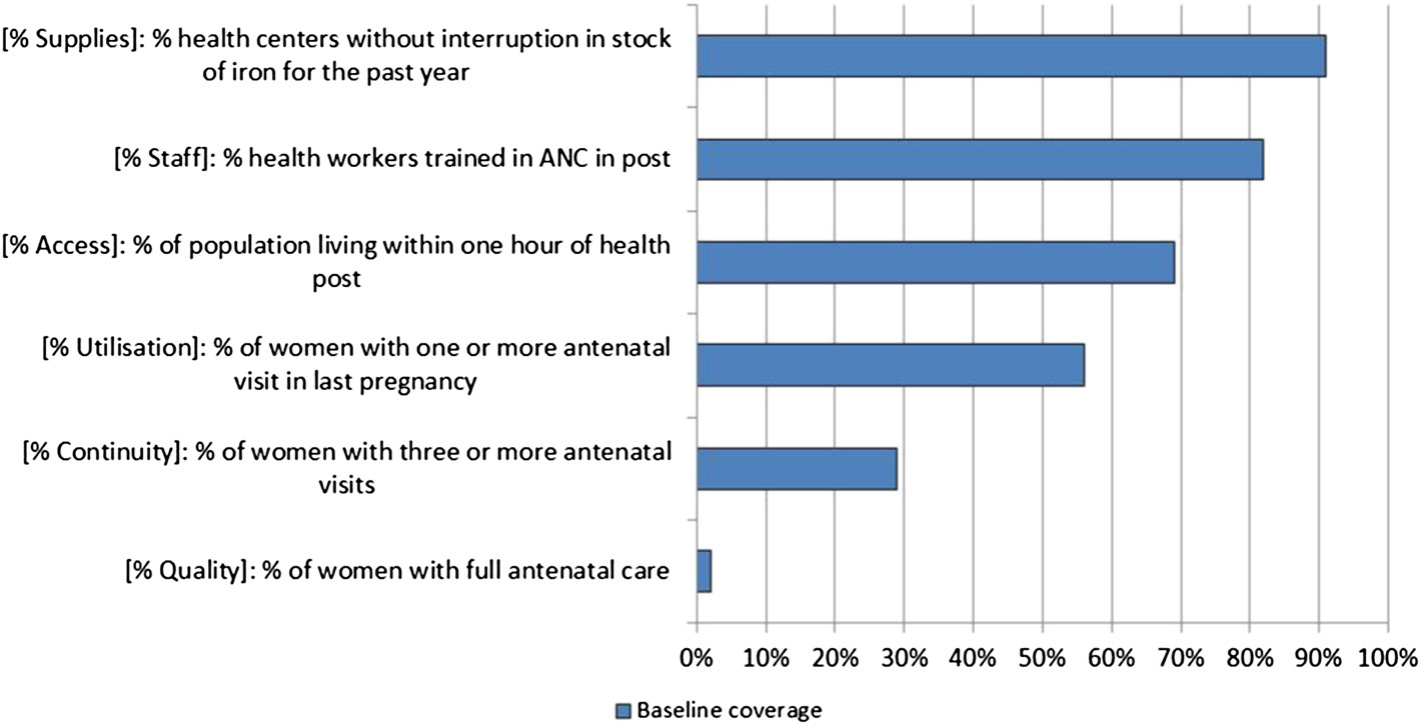
Bottlenecks chart – Antenatal Care - Hills cluster, Nepal.

Problem solving workshops with key stakeholders at different levels of the health system were undertaken to identify the root cause of the scaling-up constraints in disadvantaged locations, formulate pragmatic strategies to address them and set realistic coverage targets. In order to facilitate the discussions at the problem solving workshops, we adopted the bottlenecks framework originally developed by Tanahashi [9] and further refined by UNICEF and the World Bank [10]. This framework helps policymakers to systematically unpack the constraints to six determinants of health coverage all of which inhibit the ability of the system to increase the uptake of priority interventions. These constraints are classified according to supply (physical access to health services, availability of human resources, and availability of critical inputs), demand (first use of health services, and continuous use) and quality. Health interventions are seldom delivered in isolation. These determinants are thus examined for twelve interventions that act as proxies for other health services sharing a similar platform of delivery. Figure 1 shows the example of a bottlenecks chart used to identify health system constraints for Antenatal care in the Hills Cluster of districts, Nepal.

Results from the problem solving workshops have been validated by expert groups in all countries, particularly to ensure the feasibility of strategies and increased coverage targets within the wider regulatory environment. The extent to which these strategies were incorporated into the plans and budgets in our study sites is detailed elsewhere [7].

#### Estimating costs and impact – the decision--support model

A cohort model was developed for this project to estimate the expected costs and impact of scaling-up strategies. The supplementary material includes a brief overview of the approach with conceptual diagrams of the basic model structure (Additional file 1). The full methods and calculations of the model are described in detail elsewhere and are available as unpublished observations upon request. In brief, the model tracks an annual cohort of pregnant women until birth and follows the live births until age 5.0 years.

The model incorporates coverage, efficacy, and costing data for the 66 interventions involved in this analysis for which there are global estimates of their impact on the burden of maternal, newborn, and child mortality [11]. These interventions are organised into four phases (pre-pregnancy, pregnancy to birth, birth to age 1 month, age 1 month to 5 years) along the continuum of care starting with family planning and pregnancy-related interventions through to immunisation and curative health services for under fives. The population at-risk at the start of each phase is dependent on the coverage of interventions in the preceding phase. The methods for the estimation of intervention impact (% reduction in maternal, neonatal and under-five mortality) were adapted from the Lives Saved Tool (LiST) [12]. Deaths are first distributed into the major defined causes, then reduced by the cause-specific effectiveness (efficacy x marginal increase in coverage) of each intervention. Where more than one intervention targets a specific cause of death, impact is calculated sequentially to prevent over-estimation of deaths prevented.

Methods for modelling the coverage targets generated in the problem solving workshops and costing the strategies associated with these targets were adapted from the Marginal Budgeting for Bottlenecks (MBB) tool [10]. The costs and benefits of new strategies to scale-up MNCH interventions are calculated by comparing the new strategy to the current programme (status quo) in the population of interest.

The objective of the modelling process in the context of this study is to support decentralised decision-making for health planning and budgeting in low-income settings. For these purposes, the model aims at providing policymakers with the information required to assess the relative merits and costs of the scaling-up strategies discussed during the workshops. The model thus estimates financial rather than economic costs, which can then be used to produce sub-national budgets. To facilitate the understanding of budgetary implications, annual recurrent costs are assumed to be the same in each of the next five years. Capital costs are not annualised and are assumed to be incurred in the first year of the programme. Modified costing calculations provide both total and incremental costs. Costs are produced at baseline which allows policymakers to verify results when compared to actual expenditure. Both direct inputs into MNCH services and more general health system improvements are included in the costing calculations. As the burden of mortality in targeted areas is high, and the efficacy of most interventions on morbidity are not well established, the model only considers deaths prevented rather than other measures of health benefit. The primary outcome measures used to compare alternative strategies are the financial cost per death averted and the financial cost per capita. The time horizon of the model is a single budgeted year so future costs and effects are undiscounted.

The model was developed in Excel [13] as it was critical to have a transparent model developed in software familiar to sub-national partners engaged in the process. There is a logical stepwise progression through the model from coverage to impact and costs (see Webfigures 2–5 in Additional file 1). Inputs have been standardised and the design is fully customisable. For example, users can: (i) specify which level of coverage local estimates refer to i.e. initial utilisation, continuous coverage, effective quality coverage; (ii) define up to six health service delivery levels; (iii) organise the way interventions are grouped for the purposes of bottleneck analysis and coverage target setting; (iv) add new user-defined cost items; and (v) customise the budget to reflect local terminology and cost categories.

Due to the complexity of an analysis that combines qualitative and quantitative methods for an entire MNCH programme, we were unable to do a traditional uncertainty analysis. The model instead includes a built-in facility for univariate sensitivity analysis for a small number of important parameters. For example, the expected increase in intervention coverage is highly uncertain, yet has a large influence on costs and numbers of deaths prevented. For the purposes of this analysis, uncertainty ranges therefore reflect the sensitivity of the results to a −/+ 10% change in the intervention coverage targets.

The results of the country Investment Cases are discussed below. Tables 2, 3, 4 and 5 present causes of death, summaries of key strategies, and results for each of our study sites by country at the end of the five-year timeframe of the analysis. Costs are presented as marginal (additional) per capita costs of strategies in United States Dollars (USD), by early capital (one-off) costs and annual recurrent costs. Impact of scenarios are reported as reductions in neonatal mortality rate (NMR), under-five mortality rate (U5MR), and maternal mortality ratio (MMR) with point estimates and upper and lower bounds calculated assuming −/+ 10% of intervention coverage targets determined by policymakers.

**Table 2:** Results of impact and cost of investment case analysis in Philippines

	**Key causes of death**		**Impact (% Reduction over 5 years)**			**Cost ($US)**	
	**Maternal**	**Under-5**	**Maternal mortality ratio**	**Neonatal mortality rate**	**Under-5 mortality rate**	**Annual marginal recurrent cost (per capita)**	**First year capital cost (per capita)**
Pasay City	Post-partum Haemorrhage (34%), Ante-partum Haemorrhage (33%), Hypertension (33%)*	Neonatal Sepsis (20%), Preterm birth (15%), Congenital Abnormalities (15%), Pneumonia (11%) §	13% (11%-15%)	5% (4%-6%)	5% (4%-7%)	$0.73 ($0.61-0.92)	$0.05
			Key Strategies: Improved regulation and engagement with private sector, review of health facility reimbursement practices, training of clinical staff in family planning, IMCI, nutrition and monitoring and evaluation procedures, training in emergency neonatal care for private providers, revitalisation of community health teams to actively provide routine health services and health promotion, and improved commodity supply				
			13% (12%-16%)	5% (4%-6%)	5% (4%-7%)	$1.29 ($1.19-1.49)	$0.74
			Facility Construction Scenario Strategies: As above, with additional construction of 2 public Lying-In clinics				
Northern Samar	Post-partum Haemorrhage (64%), Hypertension (18%), Ante-partum Haemorrhage (9%), Sepsis/Infection (9%) †	Pneumonia (19%), Diarrhoea (10%), Neonatal Sepsis (10%), Preterm birth (8%), Congenital Abnormalities (8%) §	39% (32%-46%)	25% (20%-29%)	17% (14%-19%)	$2.20 ($2.01-2.40)	$2.72
			Key Strategies: Training of clinical staff in IMCI, nutrition and essential maternal and newborn care, establishment of community health teams to actively provide routine health services and health promotion, establishment of insurance membership services, campaign for facility based delivery including monitoring of compliance with applicable regulations, upgrading of hospital and primary health care facilities, recruitment of additional midwives and improved commodity supply processes				
Eastern Samar	Hypertension (33%), Sepsis/Infection (28%), Post-partum Haemorrhage (22%), Ante-partum Haemorrhage (17%) ‡	Neonatal Sepsis (16%), Pneumonia (14%), Congenital Abnormalities (13%), Preterm birth (12%), Diarrhoea (7%) §	45% (40%-50%)	26% (23%-28%)	20% (18%-22%)	$5.15 ($4.70-5.44)	$7.12
			Key Strategies: Training of clinical staff in essential maternal and newborn care, establishment of community health teams to actively provide routine health services and health promotion, establishment of insurance membership services, campaign for facility based delivery including monitoring of compliance with applicable regulations, upgrading of hospital and upgrading and construction of primary health care facilities, recruitment of additional midwives and improved commodity supply processes				

* Source: (2) Pasay City Health Office. Pasay City Vital Statistics. Manila, Philippines: Department of Health2008.

† Source : (9) North Samar Provincial Health Office. Annual Report. Manila, Philippines: Department of Health2008.

‡ Source: (10) East Samar Provincial Health Office. Maternal Death Review. Manila, Philippines: Department of Health2009.

§ Source: (17) Provincial Estimate Department of Health- National Epidemiology Center. The 2004 Philippine Health Statistics. Manila: Department of Health; 2004.

Results are based on point estimates and ranges calculated assuming −/+10% of intervention coverage determined by policymakers.

**Table 3:** Results of impact and cost of investment case analysis in Indonesia

	**Key causes of death**		**Impact (% Reduction over 5 years)**			**Cost ($US)**	
	**Maternal**	**Under-5**	**Maternal mortality ratio**	**Neonatal mortality rate**	**Under-5 mortality rate**	**Annual marginal recurrent cost (per capita)**	**First year capital cost (per capita)**
Sikka District	Hypertension (25%), Post-partum Haemorrhage (20%), Sepsis/Infection (16%), Ante-partum Haemorrhage (13%)*	Pneumonia (15%), Diarrhoea (12%), Malaria (12%), Preterm birth (11%), Birth Asphyxia (10%)†	24% (17%-28%)	14% (10%-17%)	7% (5%-11%)	$1.63 ($1.53-1.76)	$1.64
			National Priority Scenario Strategies: Infrastructure upgrade for basic and comprehensive emergency obstetric and neonatal care (BEONC/CEONC), recruitment, training and retention of staff in remote areas, coordination for adequate commodities, community participation for facility-based delivery, monitoring and evaluation activities				
			28% (22%-32%)	17% (13%-20%)	13% (9%-16%)	$3.33 ($3.23, 3.45)	$1.74
			Full Scenario Strategies: As above plus revitalisation of the Integrated Village Health Post, training of community health workers on signs of pneumonia, use of Oral Rehydration Therapy (ORT), Insecticide Treated Nets (ITN), additional training for primary health care workers, implementation of ‘Clean and Healthy Lifestyle’ in selected villages				
Merauke District	Hypertension (25%), Post-partum Haemorrhage (20%), Sepsis/Infection (16%), Ante-partum Haemorrhage (13%)*	Malaria (19%), Diarrhoea (16%), Pneumonia (15%), Birth Asphyxia (15%), Preterm birth (10%) †	35% (29%-40%)	33% (28%-37%)	13% (11%-15%)	$4.29 ($4.14, 4.37)	$1.57
			National Priority Scenario Strategies: Infrastructure upgrade for BEONC/CEONC, recruitment, training and retention of staff in remote areas, generous allowances for all midwives in the district, contract outreach teams to remote areas, voucher system to cover the cost of transport for pregnant women, coordination for adequate commodities, community participation for facility-based delivery, monitoring and evaluation activities				
			36% (29%-40%)	34% (29%-38%)	25% (21%-29%)	$7.06 ($6.91-7.21)	$2.18
			Full Scenario Strategies: As above plus revitalisation of the Integrated Village Health Post, training of community health workers on signs of pneumonia, use of ORT, ITN, additional training for primary health care workers, implementation of ‘Clean and Healthy Lifestyle’ in selected villages				
Pontianak City	Hypertension (25%), Post-partum Haemorrhage (20%), Sepsis/Infection (16%), Ante-partum Haemorrhage (13%)*	Diarrhoea (17%), Pneumonia (14%), Preterm birth (11%), Birth Asphyxia (11%), Neonatal Sepsis (5%) †	15% (6%-22%)	12% (7%-17%)	5% (3%-10%)	$0.90 ($0.73-1.17)	$0.24
			National Priority Scenario Strategies: Upgrading of health facilities for CEONC, consultation with private sector on referral and CEONC procedures, training public and private midwives in all critical Maternal, Newborn and Child Health (MNCH) interventions including immunisation, monitoring of private midwives by Midwives Association, active case finding for immunisation, media campaign for immunisation, counselling for health staff on legal protections associated with adverse events of immunisation				
			17% (8%-24%)	12% (7%-17%)	9% (5%-13%)	$1.44 ($1.31-1.73)	$0.27
			Full Scenario Strategies: As above plus activities to encourage breastfeeding (including regulation of breast-milk substitutes), revitalisation of integrated health post, training of community health workers on signs of pneumonia, use of ORT, ITN, implementation of ‘Clean and Healthy Lifestyle’, partnerships with pharmacies for delivering health messages, and to refer complicated deliveries				
Tasikmalaya City	Hypertension (25%), Post-partum Haemorrhage (20%), Sepsis/Infection (16%), Ante-partum Haemorrhage (13%)*	Birth Asphyxia (13%), Pneumonia (6%), Diarrhoea (5%), Preterm birth (5%), Neonatal Sepsis (5%)†	14% (7%-22%)	15% (9%-20%)	7% (4%-11%)	$0.77 ($0.72-0.93)	$0.36
			National Priority Scenario Strategies: Infrastructure upgrade for additional CEONC, incentives to private midwives on submission of monthly reports, recruitment and training of midwives, monitoring and evaluation particularly at primary health care level, coordination between health levels for referral of high risk deliveries, Mother’s Groups and use of MNCH books, incentives to traditional birth attendants who refer or partner with midwives				
			16% (7%-23%)	16% (10%-21%)	10% (6%-12%)	$1.11 ($1.04-1.21)	$0.44
			Full Scenario Strategies: As above plus revitalisation of integrated health post, training of community health workers on signs of pneumonia, use of ORT, ITN, implementation of ‘Clean and Healthy Lifestyle’, additional coordination and laboratory staff				

* Source: (18) National estimate. Ministry of Health. Survey Kesehatan Rumah Tangga Tahun 2001 (Household Health Survey). Report. Jakarta: Badan Penelitian dan Pengembangan Kesehatan2001.

† Source: National Institute for Health Research and Development, Indonesia (NIHRD), Basic Health Research National Report 20072008. Provincial estimate based on National estimate from (19).

Results are based on point estimates and ranges calculated assuming −/+10% of intervention coverage determined by policymakers.

**Table 4:** Results of impact and cost of investment case analysis in Orissa (India)

	**Key causes of death**		**Impact (% Reduction over 5 years)**			**Cost ($US)**	
	**Maternal**	**Under-5**	**Maternal mortality ratio**	**Neonatal mortality rate**	**Under-5 mortality rate**	**Annual marginal recurrent cost (per capita)**	**First year capital cost (per capita)**
Kendrapara	Post-partum Haemorrhage (28%), Sepsis/Infections (11%), Unsafe Abortion (10%), Ante-partum Haemorrhage (9%)*	Preterm birth (17%), Diarrhoea (16%), Pneumonia (16%), Neonatal Sepsis (15%), Birth Asphyxia (13%)‡	34% (30%-38%)	35% (33%-38%)	23% (21%-26%)	$1.61 ($1.61-1.63)	$1.70
			Key Strategies: renovation and construction of sub-health centres, upgrading of emergency maternal and neonatal care facilities, additional training for staff on postnatal care, performance incentives and travel/hardship allowances for staff, workforce planning, supervision and monitoring, ensuring supply of buffer drug stocks, community promotion activities				
Rayagada	Anaemia (24%), Post-partum Haemorrhage (17%), Sepsis/Infection (17%), Hypertension (14%) †	Diarrhoea (18%), Pneumonia (17%), Preterm birth (16%), Neonatal Sepsis (14%), Birth Asphyxia (12%)§	28% (23%-33%)	35% (32%-38%)	25% (22%-27%)	$3.92^#^	$3.56
			Key Strategies: as above				

* Source: (20) Estimate for EAG states. Sample Registration System, Office of the Registrar General of India. Maternal Mortality in India: 1997–2003: Trends, Causes and Risk Factors. 2006:1–40.

† Source: (21). Data for tribal areas in Orissa and Jharkhand. Barnett S, Nair N, et al. A prospective key informant surveillance system to measure maternal mortality – findings from indigenous populations in Jharkhand and Orissa, India. BMC Pregnancy and Childbirth 2008;8(6):1–8.

‡ Source: (15) State estimate. International Institute for Population Sciences (IIPS). National Family Health Survey (NFHS-3) 2005–06, India. 2007;1(1–540).

§ Source: (15) Tribal regions estimate. International Institute for Population Sciences (IIPS). National Family Health Survey (NFHS-3) 2005–06, India. 2007;1(1–540). # Due to large populations per capita values vary little (not reflected in $US to 2 decimal points)Results are based on point estimates and ranges calculated assuming −/+10% of intervention coverage determined by policymakers.

**Table 5:** Results of impact and cost of investment case analysis in Nepal

	**Key causes of death**		**Impact (% reduction over 5 years)**			**Cost ($US)**	
	**Maternal**	**Under-5**	**Maternal mortality ratio**	**Neonatal mortality rate**	**Under-5 mortality rate**	**Annual marginal recurrent cost (per capita)**	**First year capital cost (per capita)**
Terai cluster	Hypertension (21%), Post-partum Haemorrhage (28%), Unsafe Abortion (7%), Ante-partum Haemorrhage (6%) *	Pneumonia (20%), Birth Asphyxia (10%), Preterm birth (9%), Neonatal Sepsis (8%) †	23% (17%-28%)	39% (35%-43%)	18% (16%-19%)	$1.77 ($1.76-1.77)	$1.69
			District Cluster IC Strategies: Community based education and promotion by Female Community Health Volunteers (FCHV), additional training for staff on family planning, breastfeeding and immunisation, upgrading health posts into primary health care centres, increased staffing to enable 24 hr facilities, capacity building for local logistical management, introduction of pneumococcal and Pentavalent vaccines, introduction of community based neonatal care				
			31% (27%-35%)	46% (42%-49%)	20% (19%-22%)	$2.76 ($2.75-2.77)	$9.02
			NHSPII Strategies: As above with additional NHSPII targets for coverage and infrastructure				
Hills cluster	Hypertension (21%), Post-partum Haemorrhage (28%), Unsafe Abortion (7%), Ante-partum Haemorrhage (6%) *	Pneumonia (22%), Birth Asphyxia (17%), Preterm birth (16%), Neonatal Sepsis (13%) †	34% (30%-38%)	57% (53%-61%)	33% (31%-36%)	$2.03 ($1.98-2.00)	$0.72
			District Cluster IC Strategies: Community based education and promotion by FCHV, additional training for staff on family planning, breastfeeding and immunisation, upgrading health posts into primary health care centres, increased staffing to enable 24hr facilities, capacity building for local logistical management, introduction of pneumococcal and Pentavalent vaccines, introduction of community based neonatal care				
			40% (36%-44%)	62% (58%-66%)	36% (33%-38%)	$2.42 ($2.18-2.46)	$3.65
			NHSPII Strategies: As above with additional NHSPII targets for coverage and infrastructure				
Mountains cluster	Hypertension (21%), Post-partum Haemorrhage (28%), Unsafe Abortion (7%), Ante-partum Haemorrhage (6%) *	Pneumonia (32%), Birth Asphyxia (15%), Preterm birth (14%), Neonatal Sepsis (12%) †	26% (19%-32%)	40% (30%-49%)	24% (17%-29%)	$3.65 ($3.56-3.67)	$2.16
			District Cluster IC Strategies: Community based education and promotion by FCHV, additional training for staff on family planning, breastfeeding and immunisation, upgrading health posts into primary health care centres, increased staffing to enable 24hr facilities, capacity building for local logistical management, introduction of pneumococcal and Pentavalent vaccines, introduction of community based neonatal care				
			40% (35%-45%)	57% (50%-64%)	31% (26%-36%)	$4.20 ($4.07-4.28)	$4.02
			NHSPII Strategies: As above with additional NHSPII targets for coverage and infrastructure				

* Source: (22) National Estimate. Suvedi BK, Pradhan A, Barnett S, Puri M, Chitrakar SR, Poudel P, et al. Nepal Maternal Mortality and Morbidity Study 2008/2009: Summary of Preliminary Findings. Kathmandu, Nepal.: Family Health division, Department of Health Services, Ministry of Health, Government of Nepal.2009.

† − Source: (13) eco-region estimates for post neonatal causes (not cluster specific), Ministry of Health and Population, New ERA, ORC Macro International Inc. Nepal Demographic and Health Survey 20062007.

(23) national estimate for neonatal causes. WHO. World Health Statistics 2010. Geneva2010.Results are based on point estimates and ranges calculated assuming −/+10% of intervention coverage determined by policymakers.

## Results and discussion

### Philippines

The Philippines is a highly decentralised country with a growing urban population increasingly served by private providers, and a rural population relying primarily on the public sector. Progress toward the provision of equitable health services has been slow [14], despite the 1995 national mandate aimed at achieving universal coverage through social insurance [15]. One key factor is that access to social insurance benefits is restricted to services provided by facilities accredited with the Philippine Health Insurance Corporation as part of the quality assurance process. The factors constraining access to these accredited facilities differ between urban and rural sites, and different strategies and investments are required to scale up these services equitably.

The recent priority of the national government towards improving facility-based delivery is reflected in the focus on constraints and strategies for services during pregnancy and birth in all three sites. Prior to the Investment Case analysis, government officials in Pasay City were exploring the option of constructing two new public delivery facilities that would meet requirements for insurance accreditation, to increase the availability of quality services. The Investment Case analysis revealed an alternative that would substantially reduce the expenditure necessary to improve coverage of quality services. While public facilities providing delivery care are limited, there are several private maternity and delivery care providers, and a high proportion of women (86%) deliver in a facility. Construction of public delivery facilities would incur substantial costs, but would not add much value to alternative strategies focused on working more strategically with existing private sector providers (see Table 2). City officials can exert greater influence on private providers by enacting city ordinances that require private delivery facilities within their jurisdiction to secure insurance accreditation as a requirement to hold a business licence, improving both access for the disadvantaged and quality as a whole. Such legislative measures can be complemented by other activities targeting the quality of care provided within the private sector and demand-side information barriers preventing access by the poor.

In Pasay City these strategies focusing on the scaling up coverage of quality delivery care can be expected to achieve reductions in maternal mortality of 13% (11%-15%), newborn mortality of 5% (4%-6%), and child mortality of 5% (4%-7%) by 2015, with annual recurrent costs of USD 0.73 (USD 0.61-0.92) and early capital cost of USD 0.05 per capita. This compares favourably with the alternative scenario involving public facility construction, which would entail a much higher early capital cost (USD 0.74 per capita) without achieving an additional impact on mortality (see Table 2).

By contrast, the limited availability of facility-based services was identified as a major constraint in the mainly rural provinces, with 19% and 34% of deliveries taking place in facilities in Northern Samar and Eastern Samar respectively. Therefore strategies were aimed at getting the basics right, including strengthening procurement, training health workers, and recruiting additional midwives. A key strategy identified was the upgrading of public health facilities to provide quality maternity and delivery care, including emergency obstetric care. These upgrades would enable facilities to become accredited by the national health insurance programme, which would reduce financial barriers for the poor. The establishment of community health teams and member services for the national health insurance programme aim to raise community awareness of the programme and assist existing members in accessing their entitlements.

These combined strategies in Northern Samar aimed at improving uptake of quality delivery care can achieve mortality reductions of 39% (32%-46%), 25% (20%-29%), 17% (14%-19%), and for maternal neonatal, and under-five mortality respectively, at an annual recurrent cost of USD 2.20 (USD 2.01-2.40) per capita (early capital expenditure of USD 2.72). As a result of a more extensive infrastructure strategy in Eastern Samar – which includes building, equipping, and staffing two new health facilities, and the recruitment of a greater number of midwives to accommodate the more widely dispersed population – the per capita costs in this province is substantially greater than in Northern Samar (see Table 2) Note that the lower baseline coverage of interventions and higher levels of mortality in the Provinces mean greater mortality reductions are possible in Eastern and Northern Samar compared to Pasay City.

### Indonesia

Similar to the Philippines, Indonesia is a decentralised country pursuing universal coverage through mandatory social insurance, with a highly urbanised population served by a mixture of public and private providers. Inequity is an important concern in Indonesia. An in-depth analysis of equity in maternal, newborn and child health (MNCH) service access and health outcomes has demonstrated large variation in outcomes and intervention coverage between regions and between different population groups. In particular disparities appear to be pronounced between island groups and following the years of the decentralisation reforms in Indonesia [16].

Like the Philippines, the root causes of poor quality health services identified in all four locations differed along rural–urban lines. While in the rural sites geographical access to MNCH interventions was the key concern, in the urban areas a large and unregulated private sector compromised the quality of care. This was particularly evident in the emergency interventions where access to basic and comprehensive emergency obstetric care was found to be between 98-100% in the cities compared to 13%-46% in the districts. However, in the cities only a fraction of those with access were receiving quality care, with overall quality coverage for basic emergency obstetric care only slightly higher (25%-29%) than in the districts (18%-19%).

Many of the strategies employed in the cities to improve quality care involved not only improvement in public facilities but also stronger regulation and incentives for improvement of quality in the private sector. A clearly identified constraint in both cities was that of private midwives not following protocols for antenatal care, monthly reporting, and referral of complicated deliveries. Incentives, training, and monitoring of private midwives by the Midwives Association were therefore included as key strategies. Since emergency obstetric interventions address many of the major causes of maternal and neonatal death, the full reduction in mortality of 15% (6%-22%), 12% (7%-17%) and 5% (3%-10%) in maternal, newborn and under-five mortality respectively, in Pontianak City (with similar results for Tasikmalaya City) will only be realised if these strategies succeed in improving access to full comprehensive emergency obstetric care.

Key strategies to improve coverage of quality services in the Indonesian rural districts focused on strengthening essential components of the health system. In line with those discussed in the Philippines, these included upgrading facilities; recruitment, training, and retention of staff in remote areas; and improving demand for services.

In Indonesia, policymakers at central level also requested the development of alternative scenarios that would provide more guidance on priority setting to local government officials. We modelled a “Full scenario” scaling up the entire programme of health interventions currently delivered, along with a “National Priority scenario” that reflected the national priorities and so focused on strategies to scale up those interventions addressing the key causes of maternal and neonatal death (see Table 3). This National Priority scenario – focusing on antenatal care and emergency maternal and newborn interventions – included strategies essential to the provision of high-quality care and was seen as a better investment in three out of the four sites. For instance in Sikka district, implementation of priority strategies suggest a marginal reduction of 24% (17%-28%), 14% (10%-17%) and 7% (5%-11%) in the MMR, NMR and U5MR respectively. Annual recurrent costs of this scenario would be USD 1.63 (USD 1.53-1.76) per capita. The full scenario provides little additional benefit in terms of mortality impact (additional reduction of around 4%, 3% and 6% in MMR, NMR and U5MR, respectively), but incurs significantly higher annual recurrent costs (USD 3.33 per capita). Similar results can be seen in the case of Pontianak City and Tasikmalya City (Table 3). It should be noted that for Merauke in Papua Province, which has high post-neonatal mortality, there is a greater argument to implement the ‘Full scenario’ since it offers more substantial benefits for this age-group than for the other sites, with around an additional 12% in U5MR for this scenario over that of the ‘National Priority scenario’.

A key difference between the Philippines and the Indonesian analyses is the greater emphasis that local government officials in the former have placed on the funding linkages with mandatory health insurance. Since the health insurance scheme in the Philippines has been in place for longer, this might reflect lessons learnt during the last decade about constraints to accessing health insurance benefits. Notwithstanding differences in the focus of the analyses in both countries, common themes have emerged including the potential of relatively inexpensive strategies such as innovative public-private partnerships. In the Indonesian sites these included conducting regular coordination activities between public and private hospitals to facilitate referral and reporting of obstetric emergencies, establishing and distributing practice guidelines to both private and public providers and partnering with private midwives associations to provide additional monitoring and training opportunities. These types of partnerships require strong supervision and regulation of the private sector in urban areas. In disadvantaged rural areas there is a need to invest in “getting the basics right” to deliver quality maternal, newborn, and child health services.

### Orissa, India

The need for “getting the basics right” is also evident in the Investment Case analyses in Orissa, a predominantly rural and tribal state in India with very minor private health sector presence [17,18].

A significant rise in utilisation of key MNCH services – such as antenatal care and facility-based births – reflects the success of innovative demand-side financing schemes introduced by the National Rural Health Mission and supported by the State Government [19]. However this increase in use of health services has not always been accompanied by improved quality of care, which in turn is affected by basic supply constraints and availability of skilled manpower. Inadequate availability of health services across disadvantaged districts in India has been recently stressed as a major challenge for universal health coverage [20]. This challenge is particularly noticeable in Rayagada, a typical rural, tribal, and sparsely populated hilly district. Whilst 49% of births are attended by skilled birth attendants at a facility, only an estimated 20% take place at well-equipped facilities which meet Indian Public Health Standards. Kendrapara, a typical rural coastal district with a higher population density, faces some similar constraints.

Problem analysis indicated that there were underlying issues of low availability of basic infrastructure, supplies, equipment, and staff, particularly at peripheral facilities. Key strategies focused on strengthening outreach services and enabling task shifting to lower levels of the health system. This required the reconstruction or rehabilitation of at least 10-30% of sub-centres, the upgrading of select Primary Health Centres to provide basic emergency obstetric and neonatal care, and the installation of blood storage units at select Community Health Centres. In addition to these capital investments, other important strategies included: increasing travel allowances and additional training for sub-centre health workers; performance incentives for field staff delivering post-natal care; capacity building for management and supervisory activities; and arrangements to reimburse private sector specialists for providing emergency care to public patients. Although these findings were not surprising, and upgrading infrastructure and staffing have been part of previous plans, the IC analysis highlighted particular bottlenecks in implementation. One example is the need to include the cost of land acquisition for sub-centre construction, to ensure that they can be built in the villages where they will be used.

While investments in fundamental resources such as infrastructure and staff are critical, the analysis also revealed the potential for basic low-cost management strategies to supplement these investments by overcoming implementation issues. One example suggested by field workers and district officials, was to divide the geographical area covered by the sub-centre between health workers, rather than have them ”doubling-up” which is current practice. This would maximise the time spent per household during outreach and enable the efficient sharing of work.

Should the conservative coverage targets set in the bottleneck workshop be reached, reductions of 25% (22%-27%) and 23% (21%-26%) in under-five mortality, and 28% (23%-33%) and 34% (30%-38%) in the maternal mortality ratio, can be expected for Rayagada and Kendrapara respectively over a five year period (See Table 4). Importantly, reductions of around 35% may be expected for neonatal mortality in both districts. This is significant for Orissa as neonatal mortality rates have remained high over the last decade despite declining maternal mortality rates [21]. As shown in Table 4, additional per capita costs of USD 1.61 (annual recurrent) and USD 1.70 (early capital) for Kendrapara, and USD 3.92 (annual recurrent) and USD 3.56 (early capital) for Rayagada will be required to implement these strategies. The high marginal per capita costs for Rayagada reflect not only the lower baseline indicators for human resources and infrastructure, but also the challenges of providing health services in a sparsely populated district.

The basic management strategies that arose out of the problem solving analysis for Orissa were identified by peripheral workers in all countries. The case of the Nepal illustrates that national level analyses cannot capture such constraints and practical strategies. This can result in more costly and unrealistic targets.

### Nepal

While Nepal’s health policies and centralised planning support universal coverage, there is a concern that national progress may mask regional inequities – a situation that may worsen unless central plans are informed by realistic sub-national targets, priorities, and costs. The IC analysis in Nepal was performed in the context of the Nepal Health Sector Programme – Implementation Plan with prescribed national targets for both service delivery inputs and intervention coverage.

To analyse the difference between nationally prescribed and locally derived targets, two scenarios were modelled in Nepal. One scenario was informed by the analyses of constraints in disadvantaged locations in the three distinct ecological regions of Nepal (Mountains, Hills, and Terai) from our Investment Case. The other applied the national strategies and targets set by the Nepal Health Sector Programme – Implementation Plan.

There were broad similarities between key strategies in both scenarios, such as the revitalisation of the work of Female Community Health Volunteers and mothers’ groups, and the introduction of population-based health planning. Strategies from our Investment Case placed emphasis on overcoming the context specific constraints, and were more focused on specific logistical challenges such as the provision of accommodation for outreach staff in remote locations.

Many of the national coverage targets are not realistic for disadvantaged areas, with many involving at least a tenfold coverage increase within a five year period. For example the average coverage of skilled birth attendance in the disadvantaged districts of the mountains is 10%, while the national target is 60%. The more realistic targets set by peripheral workers during the problem solving workshops can be achieved at a substantially lower cost while still making an important impact on mortality (see Table 5). For an additional investment of between USD 2.75 and USD 5.82 per person (capital and annual recurrent costs), major progress towards MDGs 4 and 5 can be achieved in disadvantaged districts. For example, reductions in neonatal mortality rates of 39% (35%-43%), 57% (53%-61%), and 40% (30%-49%) can be expected for the Terai, Hills, and Mountains respectively. These costs compare favourably against the estimated USD 6.07-11.77 (capital and annual recurrent costs) required for the implementation of strategies recommended in the national health plan.

Costing differentials between these two scenarios are driven primarily by the more ambitious infrastructure targets set by the national health plan, which impose heavy demands on disadvantaged locations. For example in the populous terai, if the current national ratio of population to Primary Health Care Centre of 50,000: 1 were enforced, it would require a threefold increase in current facilities in our disadvantaged terai districts (from 38 to 114 over the next five years). Our analysis suggests that a ratio of 90,000:1 (an additional 25 facilities) would meet targets for basic emergency obstetric care, particularly if supported by additional services at the community and sub-post level.

## Conclusions

Some general recommendations from our ICs in different typologies can be made. In remote, sparsely populated, and poor rural areas, few incentives exist for private providers, so public investments to get the basics of the public health care system right are a necessity. Without these investments, innovative strategies like demand-side financing may increase use of health services with little or even negative effects on the quality of service. Even so, enforcing national policies or ratios of populations per provider may not be appropriate in such settings, where task-shifting and use of non-conventional delivery methods to increase access to services to remote populations may be required. Private supply in urban areas may be able to compensate for the lack of adequate investments in the provision of public services. Well articulated social insurance strategies can provide incentives in terms of rewards and penalties for private providers to deliver good quality services to the entire population, including the poor. The success of such strategies relies on the capacity of governments to regulate and supervise private providers to guarantee quality of healthcare. It also relies on the follow through of governments on their plans. Despite the inclusion of public-private partnership initiatives to improve facility-based delivery in the 2011 plans for Pasay City in the Philippines, subsequent plans reverted to the building of new public facilities [22].

The study has a number of limitations. The usefulness of all modelling exercises depends on the quality of data used. Measures of critical parameters, such as the distribution of causes of death, are frequently unavailable for sub-national analyses and often conflicting estimates exist at national level. Wherever possible, survey and validated health system data rather than modelled estimates have been used. In all settings data validation by local experts ensured that inputs for important parameters and any assumptions used in the analysis were both transparent and defendable. This process brings policymakers’ attention to the need for good quality data but does not eliminate the uncertainty of the results.

This analysis is based on the premise that the cost-effectiveness of the health interventions included in the analysis has already been established [11]. Most of the available evidence comes from randomised control trials outside the settings in our analysis, so the effectiveness of such interventions in our study sites might vary. In addition, global consensus on essential evidence-based interventions to reduce maternal, newborn and child mortality is subject to refinement. For example a recent review suggests a revised list of 56 effective interventions [23]. A review of this list reveals that there is substantial overlap with those included in our model, with some differences in scope and packages. However as a result of additional evidence becoming available, interventions included in this newer list, such as prophylactic administration of uterotonics for the prevention of postpartum haemorrhage and low dose aspirin for the prevention of hypertension, may need to be included in future analyses.

Some generalisability of costed strategies to sites of similar typology (and close geographic proximity may be made after validation from national experts. This is aided by the fact that the costing model reflects the structure of the health system in each country. However, care should be taken, in countries like Indonesia, where there are considerable regional variations in costings.

The impact estimates provided are restricted to maternal, newborn, and child mortality. Most strategies modelled relate to health system strengthening which are likely to also have an impact on maternal, newborn and child morbidity, and other health outcomes. Our model limits the mortality impact to interventions for which there is global consensus [11]. The impact on deaths due to ‘other’ causes, which form a significant proportion of child and maternal deaths in some of our study sites and which will also be amenable to health systems improvements, are therefore not captured. Additionally benefits have been measured only for the five year period even though long-term investments such as those in infrastructure will have impact beyond this timeframe.

Accelerated progress towards MDGs 4 and 5 as a nation may be achieved at the expense of equity, unless strategies to address local constraints in disadvantaged areas are adequately implemented and funded. The lessons learnt from the ICs in increasing coverage of quality health services to disadvantaged communities within different typologies should prime governments for rapid scale-up nationally. National strategies that do not take into consideration the special circumstances of disadvantaged areas risk disempowering local managers and may lead to a “business-as-usual” acceptance of unreachable goals. To effectively guide health service delivery at a local level, national plans should adopt typologies that reflect the different problems and strategies to scale up key MNCH interventions.

### Ethical clearance

Ethical clearance was obtained by the University of Queensland, Medical Research Ethics Committee and the University of Queensland Behavioural & Social Sciences Ethical Review Committee.

## Abbreviations

MNCH: Maternal, newborn and child health; MDG 4 & 5: Millennium development goals 4 and 5; List: Lives saved tool; MBB: Marginal budgeting for bottlenecks; NMR: Neonatal mortality rate; U5MR: Under-five mortality rate; MMR: Maternal mortality ratio; IC: Investment case.

## Competing interest

The authors declare that they have no competing interests.

## Authors’ contributions

All authors contributed to the research for this article. EJ coordinated the multi-country study. AC led the development of the model. AM and YP led the project team in Nepal. SL, AK and BA led the project team in the Philippines. LT and YW led the project team in Indonesia. PD, BV and RP led the project in India. EJ, SF, AM, ZD, AC, SL and PD wrote the first draft of this article and all authors reviewed subsequent drafts. All authors read and approved the final manuscript.

## Pre-publication history

The pre-publication history for this paper can be accessed here:

http://www.biomedcentral.com/1471-2458/13/601/prepub

## Supplementary Material

Additional file 1 **Supplementary material 1.** Decision-support tool. Click here for file

## Investment Case Team for India, Indonesia, Nepal, Papua New Guinea and the Philippines

Anna Bauze (Burnet Institute, Australia), Deni Harbianto (Gadjah Mada University, Indonesia), Samantha Hollingworth (School of Population Health, University of Queensland, Australia), Mohammad Karyana (National Institute of Health and Development, Indonesia), Murali Krishna (Public Health Foundation of India), Faozi Kurniawan (Gadjah Mada University, Indonesia), Michelle D. Macalintal (UPecon Foundation, Philippines), Paul A. Mariano (UPecon Foundation, Philippines), Kim Nguyen (School of Population Health, University of Queensland, Australia), Marian Angelika K. Panganiban (UPecon Foundation, Philippines), Johanna M. Santiago (UPecon Foundation, Philippines), Sneha Singh (Public Health Foundation of India) and Jacobe J. Villareal (UPecon Foundation, Philippines).
