# Supplementary material for: Investment case for improving maternal and child health: results from four countries
Source: BMC Public Health. 2013 Jun 21;13:601. doi: 10.1186/1471-2458-13-601 (PMC3701475; doi:10.1186/1471-2458-13-601)
Supplement: Additional file 1 — Supplementary material 1. Decision-support tool. [file 1471-2458-13-601-S1.doc]

**Supplementary material 1 – Decision-support tool**

This Excel-based model has been designed to support government decision-making for planning and budgeting of maternal, newborn, and child health programmes (see Webfigure 2). The model estimates the marginal impact and costs of implementing strategies to increase the coverage of priority interventions. The model constitutes the quantitative component of the Investment Case framework, and is applied in parallel with the qualitative problem solving component. The modelling steps are as follows:

1. Estimate causes of death and bottlenecks to coverage determinants. The model is customised to the local population by entering information about demography (population size, fertility rates, mortality rates), epidemiology (causes of death), and the local health system (the population served by up to six user-defined levels of providers). Over sixty maternal, newborn, and child health interventions are customised into groups or packages of interventions. Each package comprises interventions that are delivered through the same service delivery levels and are amenable to similar scaling-up strategies (see Webfigure 3). One intervention (the proxy) is selected to represent each package. In line with the principles of bottlenecks analysis adopted by Soucat et al (2), information is gathered and entered for six determinants of coverage for that intervention, namely % availability of commodities, % availability of human resources, % geographical access, % initial utilisation, % continuous utilisation, and % quality coverage (see Webappendix 1 Bottlenecks Approach). Using this information, the model generates charts to help evaluate potential bottlenecks to achieving high quality coverage. During the comprehensive problem solving process (Problem Solving using Bottlenecks Approach – See Webfigure 2) quality coverage targets are set for each proxy intervention. These targets are also used to estimate expected increases in coverage for other interventions in the same package on the basis that they would be affected by similar constraints. If it is considered those targets would not be reached for non-proxy interventions within the package (for instance because the strategies do not directly affect their delivery) override coverage targets for the relevant interventions can be applied (see Webfigure 3).
2. Estimate marginal impact of scaling-up strategies. The model uses a synthetic cohort of women based upon baseline demographics, and tracks their associated births until age five years (See Webfigure 4). Cohorts are tracked through each phase of the continuum of care, with the population-at-risk in each phase dependent on the effectiveness of interventions in the preceding phase. Effectiveness is estimated on the basis of the marginal increase in coverage, and the efficacy of each intervention against a specific cause of death. This can be adjusted to reflect partial efficacy for those women and children who do not receive full quality coverage. The impact of multiple intervention coverage on mortality due to a particular cause of death is calculated using the residual impact method, where subsequent interventions are only able to prevent deaths that have not already been prevented by previous interventions. The overall impact on a particular cause of death is then applied to the proportion of deaths attributable to that cause in the baseline. This is then used to adjust the maternal mortality ratio (MMR), the neonatal mortality rate (NMR), and the under-five mortality rate (U5MR)[[1]](#footnote-2) to represent the new situation.
3. Estimate marginal costs. The model estimates annual financial costs including intervention specific and health system related costs (see Webfigure 5). Intervention specific costs are defined as items for which the total number of required units increases with each additional user and a linear function is assumed for their calculation. The number of required units is estimated on the basis of the new intervention coverage targets and can be overridden and/or adjusted to include buffer stock requirements. Distribution and wastage factors can also be used to adjust the cost of commodities if required. Health System costs are calculated based on the number of health system providers at each level or delivery platform of the health system. We acknowledge the non-linearity of health systems costs and lack of evidence about the appropriate production function in our study countries. Therefore, the required additional number of units for items such as infrastructure, is established by policymakers when defining scale-up strategies. Estimated health system costs include both early capital expenditure and annual recurrent costs. In order to demonstrate the budgeting implications of the corresponding strategies, capital costs are presented as a lump sum in year one. Annual recurrent costs for both intervention-specific and health-system components represent the upper estimate, based on year five coverage targets.

**Webfigure 2: Overview of the Decision-support tool framework**

New Strategies defined

defined

Problem Solving using Bottlenecks Approach

New Coverage targets for proxy interventions

Impact Calculation (Webfigure 4)

Cost Calculation (Webfigure 5)

Coverage Calculation (Webfigure 3)

Inputs

Demography

Epidemiology

Coverage

Efficacy

Costs

Health System

**Webfigure 3: Coverage flowchart demonstrating how Proxy coverage determines intervention coverage.**

Coverage at Health System Levels (Baseline)

Level 1

Level 2

Level 3

Level 4

Level 5

Level 6

Intervention Coverage (Baseline)

Delivery Package for intervention at that level

Proxy 1

Proxy 2

Proxy 3

Proxy 4

Proxy 5

Proxy 6

Bottleneck Analysis for Proxy Intervention

Coverage at Health System Levels (Scenario)

Level 1

Level 2

Level 3

Level 4

Level 5

Level 6

Intervention Coverage (Scenario)

Intervention Bottleneck Determinants

% Supplies

%Staff

%Access

%Utilisation

%Continuity

%Quality

*Proportionate Increase*

*New coverage at other delivery levels.*

*Note that different increases in proxy interventions will result in change in proportion of intervention coverage delivered at each level.*

Coverage Override

*If applicable*

Numerator adjustment to quality coverage

*If applicable*

**Webfigure 4: Flowchart of impact on maternal, neonatal and child mortality**  *Continuum of care for 66 Maternal and Child Health Interventions.

# Surviving Children Age 5

# Surviving Neonates

# Live Births

# Pregnant Women

Maternal Deaths

Neonatal Deaths

*Stillbirths/Miscarriages*

1-59 Month Deaths

Continuum of Care *

MMR

NMR

U5MR

Impact of Family Planning

Baseline #pregnancies

Intervention Coverage

% Utilisation

% Continuity

% Quality

Intervention Efficacy

Intervention Impact against Cause of Death

Total Impact against Cause of Death

*Partial efficacies linked to coverage as necessary*

Baseline Mortality Rates

*Impact of other interventions on this cause of death – calculated through residual impact method*

Mortality Due to COD

Cause 1

Cause 2

Cause 3

Cause ...

Scenario Mortality

*Impact of scale up of interventions on other causes of death*

*Maternal deaths may occur at any stage between the beginning of pregnancy and 42 days post birth.*

**Webfigure 5: Costings flowchart. Note that “Early Capital Costs” are not annualised**

Intervention specific costs (*commodities, cash transfers, per case incentives)*

Early capital costs

*(pre-service training, infrastructure, equipment and vehicles)*

Total annual recurrent costs

Intervention coverage

Unit cost

Number of health providers

Quantity per health provider

Quantity per user

Unit cost

Coverage dependant costs

Health system annual recurrent costs *(Salaries, Refresher Training, Allowances, incentives, Maintenance, Utilities, Monitoring & Supervision and User defined)*

1. Includes NMR [↑](#footnote-ref-2)
